# The Case for Clinical Pathways in Radiation Oncology

**DOI:** 10.3389/fonc.2013.00220

**Published:** 2013-09-05

**Authors:** John Christodouleas, Marjorie van der Pas, Joel Goldwein

**Affiliations:** ^1^Department of Medical Affairs, Elekta, Inc., Sunnyvale, CA, USA

**Keywords:** oncology, clinical pathways, EMR, protocol adherence, practice improvement

We read the article by Dr. Louis Potters et al. with great interest and we laud their team’s effort to standardize care around evidenced-based clinical pathways. In addition to improving safety and bending the cost curve, standardizing clinical practice around expert clinical pathways has several additional benefits which we would like to highlight.

First, there is a growing body of literature from clinical trial cooperative groups that suggest adherence to protocol is associated with improved outcomes. Indeed, in some cases, the benefits of protocol adherence may even be larger than the advanced technologies or novel drug being evaluated. For example, Abrams et al. (1) conducted a planned secondary analysis of RTOG 9704 trial, a phase III adjuvant chemo-radiation protocol for pancreatic adenocarcinoma in order to determine whether protocol compliance status was associated with survival. Patients with major protocol deviations had significantly lower survival even after controlling for other known confounders and study arm. Similarly, in a recent meta-analysis, radiation protocol deviations were associated with increased risk of treatment failure and mortality (2).

Second, adherence to standardized clinical pathways may also decrease the overall variability of clinical outcomes allowing research groups and clinical practices to more easily identify new treatments that actually improve care. For instance, consider TROG 02.02 a phase III trial of head and neck cancer chemo-radiation with or without tirapazamine, a hypoxic cell cytotoxin (3). Overall, the study found no evidence of improvement in local-regional control in patients who received tirapazamine. However, secondary analyses suggested a small benefit to the experimental drug, but only in the subset of patients who received radiation therapy of an acceptable standard.

Third, clinical pathways would also promote standardization of the terminology used to describe treatments within EMRs, greatly increasing the value of EMR-based registries. There are several important nationally representative EMR-based registry efforts under way including the Oncology Data Alliance, the National Radiation Oncology Registry, and CancerLinQ. These efforts will be limited by the fact that the same treatment technique can be described in many different ways within EMRs (e.g., “VMAT” vs. “Rotational Arc”). A clinical pathways program would reduce the number of ways the same treatments are described within and across cancer programs.

As Potters et al. mention in their discussion, manufacturing and other industries have long ago embraced standardization. Since the late 1980s, medical device manufacturers have been required to comply with standards from the International Organization of Standardization (ISO) resulting in not only more consistent and predictable product quality, but also clear improvements in product safety and efficacy. Clinical pathways programs could bring the same benefits to cancer clinics and their patients. We encourage program leaders to make note of the article by Potters et al. and EMR vendors to develop functionality to support such efforts.
